# Vulcanite Base

**Published:** 1860-10

**Authors:** 


### ARTICLE III.

### *Vulcanite Base.*

FOR months past I have been using vulcanite base for mounting artificial teeth, and in every case where I have applied it, it has given satisfaction.

In making my first pieces I placed atmospheric pressure cavities similar in depth and width to those in swaged plates, and found that the suction was so great in several of them as to give pain.

Since then I have been very careful, where I use cavities, to make them very shallow. I think, however, that from a good impression the fit of the plate will be so perfect that atmospheric pressure cavities may be dispensed with altogether.

When I commenced using this work I found it difficult to keep the rubber from pressing into the joints of the teeth, and leaving a dark line upon the outside of the gums, although careful to make close-fitting joints. I now remedy this by using "Roberts' Os-artificial," or "Pierson's Osteoplastic," which, after the piece is in the flask ready for packing, I introduce into the joints from the inside and allow it time to harden.

Although no advocate for either the "Osteoplastic," or "Os-artificial," for filling teeth, yet for use in the manner above described, I think them valuable.

In the August number of the "Vulcanite Journal," dry plaster coated with a solution of glass is recommended for overcoming the same difficulty. But, after due trial, I think the preparations I have mentioned above, not only answer a better purpose, but are more easily introduced.

Another, and a more serious difficulty that I had to contend with in partial sets, where I wished to set the teeth directly against the gum, was, to prevent the rubber, when screwing the flask together, from pressing over the alveolar ridge between the teeth and gum, thereby lengthening the teeth to such a degree, by adding to the thickness of the plate, that it was necessary to grind them off, and also causing them to stand from the gum. This difficulty I remedy in the following manner.

Placing my plaster model taken from the impression of the mouth into the lower part of the iron flask, and having the teeth arranged upon a wax plate, the fac-simile of the rubber set I wish to make, and all being ready to fill the upper part of the flask with plaster, I cut away that part of the plaster model against which the gums of the teeth press, allowing them to hang free over the alveolar ridge, so that when I pour the upper part of the flask, the plaster will run under the gums of the teeth, filling up the part cut away. Separating the parts of the flask, the gums of the teeth from which you wish to keep the rubber, will be found covered, and consequently protected by plaster from any excess, which would otherwise press over them. I cut around the circumference of the piece a deep vent, and a number of small ones leading into it from the edge of the plates, as no harm results from too many vents, which would, were there not enough.

I vulcanize in one hour with the steam at 330°, by means of gas, preferring it to alcohol, the flame of which is more difficult to regulate.

I have made quite a number of pieces of this work, and so far have heard no complaint from the patients who wear them, with the exception of one who thought the plate at times produced an unpleasant taste, but upon examining his mouth I discovered a molar tooth affected with abscess and discharging pus. I extracted the tooth and have not seen the patient since.

